# Clinical-Forensic Autopsy Findings to Defeat COVID-19 Disease: A Literature Review

**DOI:** 10.3390/jcm9072026

**Published:** 2020-06-28

**Authors:** Francesco Sessa, Giuseppe Bertozzi, Luigi Cipolloni, Benedetta Baldari, Santina Cantatore, Stefano D’Errico, Giulio Di Mizio, Alessio Asmundo, Sergio Castorina, Monica Salerno, Cristoforo Pomara

**Affiliations:** 1 Department of Clinical and Experimental Medicine, University of Foggia, 71122 Foggia, Italy; gius.brt@gmail.com (G.B.); santina.cantatore@unifg.it (S.C.); 2 Department of Anatomical, Histological, Forensic and Orthopedic Sciences, Sapienza University of Rome, 00186 Rome, Italy; benedetta.baldari@uniroma1.it; 3 Department of Medical, Surgical and Health Sciences, University of Trieste, 34100 Trieste, Italy; stefanoderrico@hotmail.com; 4 Department of Law, Forensic Medicine, Magna Graecia University of Catanzaro, 88100 Catanzaro, Italy; giulio.dimizio@unicz.it; 5 Dipartimento di Scienze Biomediche, Odontoiatriche e Delle Immagini Morfologiche e Funzionali, Sezione di Medicina Legale, Università di Messina, 98122 Messina, Italy; alessio.asmundo@unime.it; 6 Anatomy, Department of Medical and Surgical Sciences and Advanced Technologies G.F. Ingrassia, University of Catania, 95121 Catania, Italy; sergio.castorina@unict.it; 7 Department of Medical, Surgical and Advanced Technologies “G.F. Ingrassia”, University of Catania, 95121 Catania, Italy; monica.salerno@unict.it

**Keywords:** COVID-19, autopsy, immunohistochemistry, post-mortem examination, forensic pathology

## Abstract

The severe acute respiratory syndrome (SARS)-CoV-2 was identified for the first time in China, in December 2019. Confirmed cases of COVID-19 have been reported around the world; indeed, this infection has been declared a pandemic. Consequently, the scientific community is working hard to gain useful information about the history of this virus, its transmission, diagnosis, clinical features, radiological findings, research and development of candidate therapeutics as well as vaccines. This review aims to analyze the diagnostic techniques used to ascertain the COVID-19 infection, critically reviewing positive points and criticism for forensic implications, obviously including autopsy. Finally, this review proposes a practical workflow to be applied in the management of corpses during this outbreak of the COVID-19 infection, which could be useful in cases of future infectious disease emergencies. Analyzing the diagnostic methods, to date, virus nucleic acid RT-PCR represents the standard method used to ascertain the COVID-19 infection in living subjects and corpses, even if this technique has several criticisms: mainly, the staff should be highly specialized, working in high-throughput settings, able to handle high workloads and aware of health risks and the importance of the results. Thus, IgG/IgM serological tests have been developed, overcoming RT-qPCR duration, costs, and management, not requiring highly trained personnel. Nevertheless, serological tests present problems; the WHO recommends the use of these new point-of-care immunodiagnostic tests only in research settings. Furthermore, nothing has yet been published regarding the possibility of applying these methods during post-mortem investigations. In light of this scenario, in this review, we suggest a flow chart for the pathologist called on to ascertain the cause of death of a subject with historical and clinical findings of COVID-19 status or without any anamnestic, diagnostic, or exposure information. Indeed, the literature data confirmed the analytical vulnerabilities of the kits used for laboratory diagnosis of COVID-19, particularly during postmortem examinations. For these reasons, autopsy remains the gold standard method to ascertain the exact cause of death (from or with COVID-19 infection, or other causes), to consequently provide real data for statistical evaluations and to take necessary measures to contain the risks of the infection. Moreover, performing autopsies could provide information on the pathogenesis of the COVID-19 infection with obvious therapeutic implications.

## 1. Introduction

The severe acute respiratory syndrome (SARS)-CoV-2 was identified for the first time in China, in December 2019: the novel coronavirus was named SARS-CoV-2, the pathogen causing COVID-19. This virus belongs to the family of viruses known to cause diseases ranging from the common cold to more serious diseases such as the Middle East respiratory syndrome (MERS) and the SARS [1,2].

The most common symptoms of COVID-19 are fever, fatigue, and dry cough. Some subjects may experience soreness and muscle pain, nasal congestion, runny nose, sore throat, or diarrhea. These symptoms are generally mild and start gradually. In the most severe cases, the infection may cause pneumonia, severe acute respiratory syndrome, kidney failure, and even death [3,4].

The incubation period is the time between infection and the onset of clinical symptoms of disease. It is currently estimated to range between 2 and 11 days, up to a maximum of 14 days [5].

Confirmed cases of COVID-19 have been reported around the world, indeed, this infection has been declared a pandemic. On the one hand, the scientific community is working hard to gain useful information about the history of this virus, its transmission, diagnosis, clinical features, and radiological findings, as well as research and development of candidate therapeutics and vaccines [6,7,8]. On the other hand, the scientific community is called on to supply fast and coordinated responses to contain the outbreak, obtaining reliable diagnostics and optimizing clinical management; this is crucial before the virus spreads and devastates weak health systems. Moreover, a critical issue is the identification of COVID-19-infected subjects, both to promptly assist them and to contain the infection, isolating potential positive subjects. Indeed, timely diagnosis, effective treatment, and future prevention are key to the management of COVID-19.

To date, the gold standard to identifying or confirming the COVID-19 infection is the reverse transcription polymerase chain reaction (rRT-PCR) on respiratory tract specimens [9,10]. Another important category of the diagnostic kit includes serological and immunological assays that largely rely on detecting antibodies produced by individuals as a result of exposure to the virus or on detection of antigenic proteins in infected individuals [10].

In this scenario there are several important concerns about the management of corpses who died “from” or “with” COVID-19 or the management of corpses who died in the period of the outbreak, particularly in the red zone of the infection. The main organizations, such as the World Health Organization (WHO) [11], Centers for Disease Control and Prevention (CDC) [12], have published documents reporting recommendations and safety strategies to adopt during confirmed and suspected COVID-19 autopsies and the safe management of corpses at the epicenter of the outbreak, however, many scientific committees’ protocols were put on hold thus stopping both clinical and forensic autopsies from being performed [13,14,15,16,17,18,19]. Each country has chosen to respond to this emergency with its own decisions; on the one hand, countries such as Italy have chosen not to perform clinical autopsies [20,21], even if recommendations to carry out autopsies had been made by the Italian scientific organization of forensic scientists [22]. On the other hand, countries such as Germany have ordered mandatory autopsies on all subjects who died with a diagnosis of the COVID-19 infection [23].

This review aims to analyze the diagnostic techniques to ascertain the COVID-19 infection, critically reviewing the positive points and limitations for forensic implications, obviously including autopsy. Finally, this review proposes a practical workflow for the management of corpses during this outbreak of the COVID-19 infection, which could be useful in cases of future infectious disease emergencies.

## 2. Methods

For this literature review two different databases (PubMed and Google Scholar) were questioned from 1 January 2019 to 15 June 2020. The main keywords “COVID-19,” “nCov 19,” “Sars Cov 2” were crossed with different terms for each paragraph. For the “COVID-19 laboratory-based molecular testing” section the specific terms used were “Molecular test,” “Real-time reverse transcriptase-PCR,” “Rapid diagnostic test,” “Serological test,” and “antibody/antigen detection.” For the “autopsy findings” paragraph the following key search terms were crossed with the main keywords “post-mortem,” “autopsy,” and “biopsy.”

The papers were selected after the evaluation of the title and abstract, selecting the studies that met the inclusion criteria. Moreover, the references of the selected articles were also reviewed.

## 3. COVID-19 Laboratory-Based Molecular Testing

In response to the growing COVID-19 pandemic, each country has adopted specific diagnostic countermeasures, consisting in laboratory-based molecular testing with the aim of identifying the specific genetic targets of the virus in different samples. Indeed, different kinds of samples could be tested showing differences in the test sensitivity, such as upper respiratory tract specimens (i.e., sputum, nasal or throat swabs), lower respiratory tract (i.e., endotracheal aspirate, bronchoalveolar lavage), and other samples (such as feces, ventricular lavage and blood). At the same time, multiple diagnostic test manufacturers are working hard to develop rapid laboratory tests based on antigen or antibody detection in blood or serum.

During the outbreak, the identification of positive subjects is very important, not only to promptly treat them but also to isolate them, using all the necessary procedures to contain the infection. For these reasons, it is essential to avoid false positive/negative results [24]. Indeed, on the one hand, a false positive result may cause severe consequences both for the subject (undergoing unnecessary treatment; moreover, the subject could be hospitalized with other true positive patients, and become infected) and for the socio-economic consequences (for example, if the subject is a health worker and cannot work); on the other hand, a false negative result could be decisive in the spread of the pandemic infection, delaying medical assistance for the subject [25].

For this reason, independently of the test used (both molecular and immunological techniques), it should be fundamental to avoid preanalytical errors. The general vulnerabilities such as a collection of inappropriate or inadequate material (both quality and volume), lack of identification, inadequate specimen procedures (collection, transport, and storage), represent the important bias of all laboratory techniques; moreover, there are the specific and analytical problems that are strictly related with the methods applied [26].

### 3.1. Detection of the Molecular Region of the COVID-19 Virus

The identification of COVID-19 subjects represents a challenge due to the rapid spread and increasing number of positive subjects. In this viral infection, nucleic acid detection-based approaches have become a reliable technology for viral detection. The polymerase chain reaction (PCR) method is considered the “gold standard” for the detection of different viruses, considering that this technique guarantees both high sensitivity and specificity [27,28]. In the case of RNA viruses, such as COVID-19, real-time reverse transcriptase-PCR (RT-PCR) is the gold standard method for diagnosis: particularly, it has several important benefits such as a well-known sensitivity and specificity; moreover, real-time RT-PCR allows to the identification of the infection in the early phase, even if there is a period of a few days, called the “window” period, where the subject is negative [29]. It is important to note that this period is shorter for molecular testing and longer for immunological technologies [30]. The essential characteristics for the RT-PCR test used for the diagnosis of the COVID-19 infection must follow the WHO recommendations [31], along with those proposed by the CDC [32]. Both documents suggest detecting the presence of the three regions of the virus; in this way, many kits have been produced around the world [33]. According to these recommendations, the molecular technique could be applied to different kinds of samples such as nasopharyngeal and/or oropharyngeal swabs and lower respiratory specimens (sputum and/or endotracheal aspirate or bronchoalveolar lavage). All samples may be used within 5 days (storing at 2–8 °C), and for more of 5 days (storing under −70 °C).

The major concerns about this method could be related to specific vulnerabilities, which could generate false positive/negative results. Sample contamination is one problem that may generate a wrong result. Obviously, it is strictly related to different phases (for example, during sampling), and it could be avoided following the prescribed procedures in the case of viral infection [34]. Moreover, as demonstrated in other viral infections such as HIV, another important problem is related to the use of antiretroviral therapy: indeed, the viral load could be insufficient to be detected, generating false negative results [35].

Analyzing the problems, as previously described, one of the most important problems is related to the “window” period: indeed, in the early period of the infection, to detect positive subjects the viral load should be over the RT-PCR threshold. For this reason, in the COVID-19 infection, a range from 0 to 5 days is necessary to ascertain positivity working with nose or throat swabs, or combination thereof. Moreover, as discussed by Lescure et al. [36], there are different clinical and biological types of evolution in COVID-19 patients, suggesting a different viral testing strategy. For example, in the late phase of the infection, it is possible to find both negative subjects with an upper airway sample and severely infected individuals continuously positive from the lower respiratory tract sample. Moreover, in the late period of the infection, it is possible to find positive results in the upper respiratory tract samples, but it is impossible to know whether these are live virions or just emitted viral RNA from dead cells that were detected in the late phase.

Another analytical problem is related to the different haplotype of COVID-19: during the replication phases it is possible to find an interindividual variability that could generate several problems in the accuracy of RT-PCR detection related to immune response [37,38,39]. Obviously, the efficacy of this method is conditioned by the quantity and the quality of the biological material. In general, the most used sample to perform the screening on COVID-19 subjects is the throat or nasal swab or their combination: this kind of sample requires that it contains sufficient amounts of viral RNA. This can be a challenge because the amount of viral RNA not only varies tremendously between subjects (it can depend on the timing of the test and/or the onset of symptoms). Moreover, this sampling technique is personnel-dependent, varying significantly from health professional to health professional. Finally, other problems can be related to the use of a non-validated assay, instrumental malfunction, and misinterpretation of data [40,41].

It is important to note that the application in post-mortem samples was previously described [42,43,44]. These studies revealed that this investigation can be useful, confirming COVID-19 positivity persistence even after death.

### 3.2. Rapid Diagnostic Tests Based on Antigen Detection

In a recent review, Bruning et al. [45] highlighted the importance of defining rapid, sensitive, and specific tests particularly in the current era of emerging novel respiratory viruses; the identification of rapid diagnostic tests could be very useful for implementing pathogen-specific infection control measures. The diagnostic tests based on antigen detection in blood or serum have the potential to fulfil these needs, but it is important to be aware of their limitations in diagnostic performance.

Usually, the rapid tests aim at detecting the presence of specific immune proteins in the tested biological fluids. These essays take on a wide range of different formats but essentially consist of an antigen or antibody, immobilized on a surface of the antibodies versus viral antigens. The samples commonly used are serum and blood: the rapid tests provide results within 30 min. In general, the antibodies against the antigen are usually fixed on a paper strip enclosed in a plastic casing, generating a detectable sign from 10 to 30 min. In the case of the COVID-19 infection different kits have been developed to identify the presence of immunoglobulin M (IgM) or immunoglobulin G (IgG) antibodies or both. It is well established that after a viral infection, IgM is the first line of defense, before the generation of adaptive, high-affinity IgG responses that guarantee long-term immunity, representing immunological memory [46]. The evaluation of the time between infection and positivity to the test is important. For example, in the SARS infection, IgM was found in blood samples from 3 to 6 days after contagion, while IgG was detected after 8 days [47]. To the best of our knowledge, Li et al. [48] reported the first rapid IgG-IgM combined antibody test for COVID-19 diagnosis. Based on their results, the reported IgG-IgM combined antibody test kit demonstrated a sensitivity of 88.66% and specificity of 90.63%. In the same study, three main limitations were described in the development of the kit: i) The impossibility to detect the antibodies (both IgM and IgG) when their levels are below the detection threshold; ii) the inter-individual variability in immune response; iii) the lability of the IgM antibody because its levels rapidly decrease and disappear about 2 weeks after infection. Moreover, the most important limitation is related to the possibility that the antibody detection tests targeting COVID-19 may also cross-react with other pathogens, including other human coronaviruses [49], generating false-positive results. Following these suggestions, a wide range of serology immunoassays (IAs) have also been developed in order to obtain a quick method for COVID-19 diagnosis [50].

The serologic reactivity to the COVID-19 virus is related to several factors: for example, the immunological activity of chronically ill older adults is worst compared to younger adults [51].

As previously described, the presence of antibodies is strictly related to the phase of infection, hypothesizing the time from onset of the illness; in other words, it is possible to define if the subject is in the early, acute or late phase of infection, or alternatively, if the subject is completely fully healed [52]. Nevertheless, the correct timing to detect IgM and IgG response after the COVID-19 infection is so far unclear, and only a few studies are available and these have divergent results [53].

Other important factors that can influence the test are preanalytical and analytical vulnerabilities. During the outbreak, the scientific community worked hard to improve rapid diagnostic tests. In a recent report, Hoffman et al. [54] reported a sensitivity of 69% for the IgM test and 93.1% for IgG, with an overall specificity of 100% and 99.2% for IgM and IgG, respectively, reporting only one false positive in the IgG test. Evaluating the same test on the PCR-positive cases as true positives, the accuracy of the test was 94.1% for IgM and 98.0% for IgG. Finally, the positive and negative predictive values for IgM were 100% and 93.2%, respectively. For IgG, the corresponding values were 96.4% and 98.4%. It is important to note that in several studies the samples were obtained from hospitalized patients. Undoubtedly, enrolling hospitalized patients represents an important bias in the evaluation of results. Indeed, these patients could have high antibody levels, showing a falsely high sensitivity and specificity, while this test should be carried out on subjects with moderate symptoms, who account for the majority of infected individuals [49,55].

These data highlighted an important limitation of these tests: such tests might miss half or more of COVID-19 infected subjects. This consideration could be linked to the subjects tested or to the specificity of the test used. It could be possible that there was a cross-reaction with other antibodies against other human coronaviruses that cause the common cold, with the risk of generating false-positive results [56,57]. Further experimentation to improve the commercial kits is necessary to reduce or eliminate the number of expensive molecular tests. Mainly, it should be imperative to achieve a high degree of diagnostic accuracy to use rapid diagnostic tests in the triage of suspected COVID-19 subjects.

Finally, the last consideration is related to the possibility of COVID-19 reinfection. However, there is currently no evidence to support this: to date, no study has been published about COVID-19 immunity [58]. This question remains open; further studies are needed to better understand this important question.

For these reasons, the WHO suggested avoiding rapid diagnostic tests, even if the same organization encourages the development of research in the same field in order to obtain an accurate kit [59]. Furthermore, nothing has yet been published regarding the possibility of applying these methods during post-mortem investigations.

## 4. Autopsy Findings

In this section, all the described patterns of the different authors are reported to delineate each aspect of the complex pathological picture.

The first two reports were published in February 2020. The first report concerning lung histological findings was published by Xu et al. [60] who reported a mini-invasive post mortem examination (tissue samples were taken from lung, liver, and heart) performed on a 50-year-old man positive at the COVID-19 rRT-PCR assay. On the lung tissue, the authors described evident desquamation of pneumocytes, hyaline membrane formation, and edema; all findings were indicative of acute respiratory distress syndrome (ARDS). Moreover, they found interstitial mononuclear infiltrate and multinucleated syncytial cells [60].

Tian et al. [61] reported two cases of COVID-19 patients who underwent lung lobectomies for adenocarcinoma. They examined only the lung biopsies describing several histological changes such as proteinaceous exudates in alveolar spaces, scattered large protein globules, intra-alveolar fibrin with the presence of inflammatory cells (mononuclear and multinucleated giant cells). Moreover, they described a diffuse expansion of alveolar walls and septa owing to fibroblastic proliferation and type II pneumocyte hyperplasia [61]. The same research group published a new study reporting the data obtained from four post mortem needle core biopsies of lung, liver, and heart of four patients who had died of COVID-19 pneumonia. They reported their previous results, suggesting the possibility of distinguishing the infection stadium evaluating the degree of fibrosis [62].

Karami et al. [63] described a case report of a pregnant patient who had died from COVID-19. In this case, they performed a partial autopsy, sampling only the lungs. They described alveolar spaces with a focal hyaline membrane, pneumocyte proliferation, and metaplastic changes. Moreover, the presence of inflammatory cells (mononuclear cells, lymphocytes, and macrophages) were described [63]. A similar pattern was recently documented in the study by Schaller et al. about autopsies on ten subjects whose positivity to COVID-19 had been collected via nasopharyngeal swab on hospital admission [64]. The histopathological findings revealed a non-uniform distribution of diffuse alveolar damage (DAD) in different phases, mostly in the middle and inferior lung lobes: exudative phase with hyaline membrane formation, intra-alveolar edema, and thickened alveolar septa, and perivascular infiltration of plasma cells; organized phase with fibroblastic proliferation and consequent parenchymal fibrosis, and type II pneumocyte hyperplasia. However, an introduction to this study is the observation of alterations affecting organs other than the lung: mild lymphocytic myocarditis; periportal plasma cell infiltration, and signs of liver fibrosis have also been described. As far as myocardial involvement is concerned, Sala et al. were the first to detect the presence of myocardial T-lymphocytic inflammation in a COVID-19-positive subject, associated with interstitial edema and limited focal necrosis [65]. However, no SARS-CoV-2 genome was detected within the myocardium, maybe related to the limitation of the bioptic procedure, as suggested by the authors [66] or due to an autoimmune systemic reaction, instead of direct virus-induced damage.

It is interesting to note that two reports described two different pulmonary patterns related to the COVID-19 infection. Based on the histological and immunohistochemical findings of five cases, Magro et al. [67] suggested that the pathophysiology of COVID-19 may be different compared to typical ARDS. The authors described the presence of systemic microvascular thrombosis. Indeed, they described significant fibrin deposition within the intra-alveolar septa and alveolar spaces, accompanied by marked hemorrhage and hemosiderin deposition, naming this pattern as atypical ARDS [67]. However, above all, this study observed that the positivity to immunohistochemical staining with antibodies to C3d, C4d, C5b-9, and MASP2, which is the basis of the ARDS atypical pattern, would involve extensive deposition of complement components within the lung septal microvasculature. Consequently, this would cause membrane attack complex-mediated microvascular endothelial cell injury activation and promotion of prothrombotic activity. It should be remembered that the vascular deposition of C5b-9 is typical in many micro-thrombotic syndromes, such as antiphospholipid antibody syndrome [68]. In the same way, Cai et al. analyzed the histological findings of seven patients who had died from COVID-19. In their case series, they distinguished two histological patterns: the first was characterized by edema, hyaline membranes, inflammation, and microthrombi; the second pattern had pauci-cellular infiltration, with lung parenchymal injury with septal capillary damage [69]. This pattern of “COVID-19 pneumonia” has recently been confirmed by Edler et al. in which 8 out of 18 cases documented the presence of fibroblasts, protein-rich exudate, and hyaline membranes as a sign of DAD, with squamous metaplasia and fibrosis being most evident in the advanced stages of the pathology [44]. However, small pulmonary arteries often showed a pronounced infiltrate of lymphocytes and plasma cells with a pattern devoid of vasculitis. To evaluate pneumocyte involvement, Suess et al. [43] used immunohistochemical staining with antibody anti-TTF1 (thyroid transcription factor-1) in their case report, which revealed severe type II pneumocyte hyperplasia with viral cytopathic-like changes affecting nucleoli and many mitotic figures. However, the most interesting finding in their autopsy was the pericardial inflammation characterized by lymphocytes and plasma cells infiltration. At the renal level, however, intact glomeruli and acute renal tubular damage have been documented. With anti-COVID-19 antibodies localized only to the renal tubular cells of the tissues infected by immunohistochemical staining [70]. Additionally, COVID-19 viral nucleocapsid protein antigen, cell apoptosis and proinflammatory cytokine expression have been documented in the spleen and lymph nodes of autopsies of infected subjects. In addition, the spleen corpuscles are atrophic, along with hyperplasia of the interstitial vessels and fibrous tissue of the septa. At immunohistochemical staining with anti-COVID-19 antibodies, positivity was found mainly in red pulp and splenic blood vessels, germinal and capillary centers, cellular cytoplasm. Brain involvement, instead, appears unclear. Some authors suggested that pan-encephalitis, meningitis, and brainstem neuronal cell damage and even CNS hemorrhage could be linked to the COVID-19 infection [71], while according to other authors, only hypoxic outcomes and not encephalitis at standard staining, nor cytoplasmic viral inclusions on immunohistochemical tests have been shown, if not at low levels in a few sections, in subjects affected by COVID-19 [72].

Moreover, two complete autopsies were reported in the study performed on COVID-19 patients by Barton et al. [73]. Even if the macroscopic investigation was performed on several organs, the microscopical investigations were carried out on lung specimens. In their final report, they described two different pictures: in one case, the histological examination reported the presence of thrombi within a few small pulmonary artery branches. Moreover, congestion of alveolar septal capillaries and edema were described. On the other hand, in the second case, no evidence of DAD was reported, even if immunohistochemistry showed similar findings to the other case. They concluded that the first case can be listed as death from COVID-19, while the second one died with COVID-19 [73].

Finally, Wichmann et al. [23] reported the German experience describing the data obtained performing 12 consecutive autopsies, starting with the first known SARS–CoV-2–positive death. At the gross examination, they described the presence of massive pulmonary embolism, with the thrombi derived from the deep veins of the lower extremities (4 cases); moreover, they reported fresh deep venous thrombosis without pulmonary embolism (3 cases) and the presence of deep venous thrombosis in all subjects. Two-thirds of the patients involved in this study had fresh thrombosis in the prostatic venous plexus. In the same way, the histological findings reported diffuse alveolar damage consistent with early acute respiratory distress syndrome (8 cases), describing the predominant presence of hyaline membranes with microvascular thromboemboli, capillary congestion, and protein-enriched interstitial edema. In other cases, they found no diffuse alveolar damage but extensive granulocytic infiltration, describing microthrombi within small lung arteries. In light of these findings, they reported that the cause of death was found within the lungs or the pulmonary vascular system [23].

As far as the thrombotic aspect is concerned, of 21 positive COVID-19 patients from the Menter et al. [74] study, the cause of death was attributed to diffuse alveolar damage in the exudative phase with capillary congestion accompanied by microthrombi despite anticoagulation. Other morphological substrates highlighted, only in some cases, were: superimposed bronchopneumonia, pulmonary embolism, alveolar hemorrhage, and vasculitis [74]. Varga et al. demonstrated a diffuse endothelial inflammation in 3 COVID-19 positive cases, among whom lymphocytic endotheliitis was noticed in lung, heart, kidney, and liver samples from 1 case, hypothesizing that endothelial dysfunction is the principal determinant of COVID-19 pathology inducing vasoconstriction with subsequent organ ischemia, inflammation, edema, and pro-coagulant state [75]. Supporting the thesis of an increased pro-coagulative state, Dolhnikoff et al. found in 8 out of 10 COVID-19-positive cases, whose samples for histological investigations were obtained by ultrasound-based minimally invasive autopsies, the presence of small fibrinous thrombi in the pulmonary arterioles in areas of both damaged and more preserved lung parenchyma and a large number of megakaryocytes in the capillaries [76]. A recent confirmation of this pathophysiological mechanism comes from the study by Ackermann et al. [77], in which seven pulmonary histological pictures of subjects who had died from COVID-19 were compared with samples from subjects who had died from influenza AH1N1. In addition to the now known widespread alveolar damage with pneumocyte-type-2 hyperplasia and intra-alveolar fibrin deposition, fibrin thrombi have been documented in pulmonary arterioles, without complete luminal obstruction and, in some cases, associated with intussusceptive angiogenesis. In the same study, it was revealed that 69 angiogenesis-related genes were differentially regulated during the COVID-19 infection. Furthermore, immunohistochemical staining via anti-angiotensin-converting-enzyme-2 (ACE2) antibodies showed ACE2-positive alveolar epithelial and endothelial cells and ACE2-positive lymphocytes in lung tissue samples. Further confirmation of the involvement of coagulopathy and thrombosis patterns has recently been reported in the study of Carsana et al. with the formation of fibrin microthrombi in the small arterial vessels (<1 mm in diameter) in the context of areas of diffuse alveolar damage associated with diffuse endothelial damage [78]. The lung tissue also presented changes of the exudative and early or intermediate of DAD, namely pneumocyte necrosis (in all cases), hyaline membrane (in 33 cases), interstitial and intra-alveolar edema (in 37 cases), type 2 pneumocytic hyperplasia (in all cases). These findings are associated with an inflammatory infiltrate, consisting of alveolar macrophages and interstitial lymphocytes. The involvement of both ascertained DAD and suggested ante-mortem disseminated intravascular coagulation patterns could explain the severe hypoxemia that characterizes ARDS in subjects with COVID-19 [79].

## 5. Discussion

To date, virus nucleic acid RT-PCR represents the most used tool to ascertain the COVID-19 infection. This technique has several criticisms: mainly, the staff should be highly specialized, working in high-throughput settings, able to handle high workloads, and aware of the health risks and the importance of the results. Furthermore, the PCR tests require certified laboratories, expensive equipment, and trained technicians. According to recent estimates, false negative results due to inadequate sampling obtained with RT-qPCR are more common than initially thought [26]. To reduce the bias related to this method, other techniques such as CT imaging, and hematological parameter analysis has been applied [80]. For these reasons, the importance of the sampling in the RT-PCR test has to be stressed: this step represents the real problem of this technique, and it should be performed by well-trained personnel. Therefore, as suggested by the WHO, nucleic acid amplification testing remains the gold standard method, because of its sensitivity and the shorter window period [81], even if the development of a rapid, simple to use, sensitive, and accurate test to quickly identify COVID-19 patients is important, both to prevent virus transmission and to assure timely treatment.

IgG/IgM serological tests offer a supplement to RT-qPCR, but cannot be used alone, because of their lower sensitivity and specificity. First of all, the simplicity of the sampling: collecting blood specimens represents well-known methods, guaranteeing fewer variations than nasopharyngeal viral RNA specimens. Moreover, the stability of human antibodies is higher compared to the viral RNA load. Finally, swabs have a high variation related to the sampled area [82]. It is important to note that the immunological tests have several limits too. For example, when suspected COVID-19 subjects are tested, their pre-existing serologic status against other coronaviruses that could generate a cross-reaction is unknown. Moreover, one of the most important problems is related to the possibility of having a prolonged viral shedding even after seroconversion [83]. Furthermore, several patients who tested positive for the COVID-19 infection on the RT-PCR, have not shown the antibody responses in the late phase of the infection [55]. For these reasons, their use is widely discussed by the scientific community, because they cannot quickly diagnose acute infection in order to determine the course of treatment. Some clinicians have used these tests to evaluate antibody responses, in order to perform a presumptive diagnosis of past COVID-19 positivity in cases where molecular testing was negative but where there was a strong epidemiological link to the COVID-19 disease [5,84].

**Figure 1:**
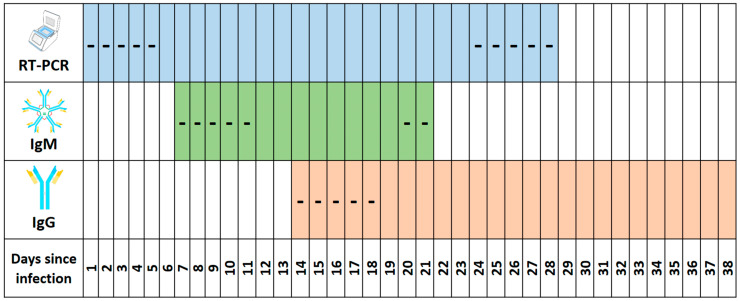
Summary of the positivity to the test used in relation to the time interval from infection: different colors are used for each analysis (blue for RT-PCR, green for IgM antibodies, pink for IgG antibodies. For each test, with the sign (-) the “window” period (number of days when the subject is negative even if he is positive, is reported); moreover, the same sign is used to indicate the last phase of the positivity for the RT-PCR and IgM tests.

Considering all these aspects, Figure 1 shows the efficacy of the test used in relation to the time from infection.

Based on all the data obtained through this review of the literature, the most important purpose of this paper is the creation of a flowchart to guide pathologists when there is an epidemic or pandemic.

**Figure 2:**
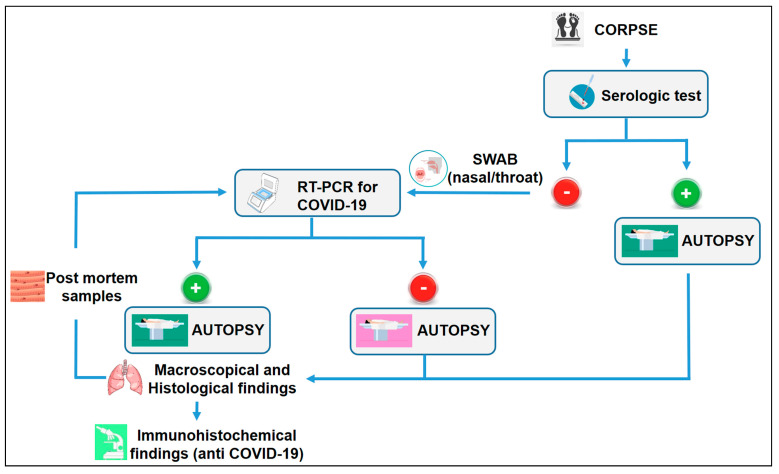
Autopsy remains the only tool to confirm or exclude the COVID-19 infection. This flowchart should be applied for all cases of death in the different areas where there is the COVID-19 infection.

As summarized in Figure 2, when a pathologist is called to ascertain the cause of death in subjects with historical and clinical findings concerning their COVID-19 status or without any anamnestic, diagnostic or exposure information, it should be mandatory to follow these suggestions.

The first step is the serological test; if the test is positive, autopsy should be performed following international recommendations [11,14,22]. To exclude false positivity, during the autopsy it should be mandatory to examine the lungs both macroscopically and microscopically (using standard staining). If the gross and microscopical findings suggest a typical lesion of the COVID-19 infection, the immunohistochemical investigation with the anti-COVID nucleocapsid antibody should be performed together with lung sampling to obtain molecular data. On the contrary, if the results of the serologic test are negative, nasal and/or throat swabs should be taken. If the result is positive, to exclude a false-positive, it is important to analyze the lung performing the gross examination, histological investigation and the immunohistochemical analysis with the specific antibodies against the virus. Moreover, the lung should be sampled to obtain molecular confirmation. However, if the swab is negative, in the presence of clinical symptoms of the COVID-19 disease, autopsy should be performed to confirm the molecular results. Particular attention should be paid in these cases, because of the above described possibility of false negatives. Therefore, autopsy has to be conducted following international recommendations established for suspected COVID-19 positive corpses. If the data obtained after the gross and histological examination suggests a suspected COVID-19 infection, it is mandatory to perform the immunohistochemical evaluation with anti-COVID-19 and further molecular investigations. It is important to highlight anti-COVID-19 immunohistochemical limitations. The standard bright field immunohistochemical technique comprises three components: slide preparation, immunohistochemical procedure, and interpretation of the results. Factors which may affect the outcome of immunohistochemical include tissue handling with the sampling, epitope retrieval, storage and handling of tissue sections, choice of antibody, detection method, and interpretation procedure [85].

In this flowchart, it is important to note that it is impossible to exclude the COVID-19 infection even if both the serological and molecular test are negative. As abundantly discussed, both methods have several pitfalls and so they could generate false positive/negative results. Based on experience in Italy, in a specific act of the Ministry of Health (Circular of General Direction of Health Prevention) [86], it is reported “for the entire period of the emergency phase, forensic or clinical autopsies should not be carried out in full-blown cases of COVID-19,” basing this consideration only on the molecular diagnosis. For this reason, during the critical phase of the outbreak, only a few autopsies were performed, leaving the diagnosis to molecular tests. On the 1st of June, the Italian Ministry of Health revised its act [87] by authorizing autopsies on people who died for/with COVID-19. After death, the swab test was usually performed on the corpse: If positive, the cause of death was listed as death from or with COVID-19, without any further differentiation; if the swab result was negative, the cause of death was listed as death without COVID-19. However, based on the results of the present review, it is not possible to exclude the positivity of the subject if the serological and swab tests are negative. It could be possible that the subject did not have the viral concentration higher than the threshold value of RT-PCR, particularly when there are problems related to the sampling. For this reason, in the flow chart, autopsy is marked in red: the pathologist should take the same precautions as for a positive case. Moreover, performing the autopsy, it could be ascertained the positivity in the “blind” case, quarantining all the people who came into contact with the victim, reducing the risks of infection.

**Figure 3:**
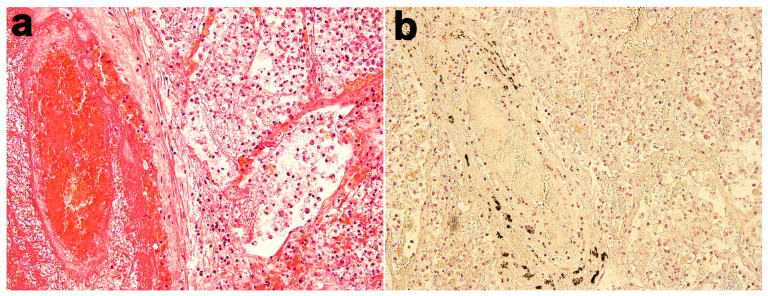
(**a**) H&E staining (20×) showing unilateral diffuse alveolar damage with a focus on a microthrombus and surrounding lung tissue characterized by severe organizing pneumonia. (**b**) Anti-COVID nucleocapsid antibody (anti Coronavirus-FIPV3-70 Santa Cruz Biotechnology, Inc, Dallas, TX, USA) positivity (data obtained and collated in the course of personal death from COVID-19 infection case, which was negative both to serological and swab tests).

In the same way, when the molecular test is positive, autopsy represents the only tool that can define the exact cause of death. The identification of death from or with COVID-19 must be performed through immunohistochemical investigation, evaluating the presence of antibodies anti-COVID-19 infection (Figure 3). For these reasons, in all post-mortem examinations, both performed for judicial and clinical purposes, both fresh and fixed samples of all organs and biological fluids should be collected to perform all necessary investigations in order to ascertain the positivity or negativity of the subject, independently from the swab or serological data.

Moreover, autopsy not only provides useful elements for the diagnosis of the cause of death, being able to establish if a subject died from or with the COVID-19 infection, with implications on statistical and global health data, but also information on pathogenesis. The correlation between the clinical picture and the pathological findings collected during autoptic exams allows the pathologist to provide evidence-based-science, fostering the ever-present Morgagni’s belief that all diseases are subtended by an alteration of an anatomical substrate [88]. However, without going too far back in time, it was only November 2019, just a few months before De Cock et al. affirmed in a perspective essay in the New England Journal of Medicine “If the dead could speak, they probably would wish to contribute to the welfare of the living. We believe it is time to establish a global research and public health agenda that focuses on learning from the dead” [89], a wish that has been disappointed to date. However, it is only through autopsies that evidence has been reached on otherwise clinical suspicions that the COVID-19 infection could manifest itself in two patterns: typical or atypical ARDS, with evident implications on therapy. Moreover, in this review we have highlighted the pitfalls related to other diagnostic methods, particularly in the post-mortem examination.

*Mortui vivos docent*!
